# Hysteroscopic Resection Followed by Adjuvant Radiotherapy: Report of a New Therapeutic Approach to Primary Cervical Diffuse Large B-Cell Lymphoma

**DOI:** 10.3390/ijerph191811779

**Published:** 2022-09-18

**Authors:** Guglielmo Stabile, Lara Sancin, Pierino Boschian Bailo, Chiara Ripepi, Andrea Romano, Stefania Carlucci, Giuseppe Ricci

**Affiliations:** 1 Institute for Maternal and Child Health-IRCCS “Burlo Garofolo”, Department of Obstetrics and Gynaecology, 34137 Trieste, Italy; 2 Department of Medicine, Surgery and Health Sciences, University of Trieste, 34127 Trieste, Italy; 3 Department of Obstetrics and Gynecology, Azienda Sanitaria Universitaria Giuliano-Isontina, San Polo Hospital, Gorizia-Monfalcone, 34149 Trieste, Italy

**Keywords:** cervical cancer, lymphoma, hysteroscopy, conservative treatment, minimally invasive approach

## Abstract

The female genital tract can be involved as a secondary manifestation of disseminated lymphomas or leukemia but can rarely be the primary site of so-called extranodal lymphomas. Primary lymphomas of the female genital tract can affect the uterine corpus, uterine cervix, vulva, vagina or adnexa. Only about 0.008% of all cervical tumors are primary malignant lymphomas. The presenting symptoms are unspecific and also refer to much more common diseases of the female genital tract. Cervical cytology is usually negative. Preoperative diagnosis requires deep cervical biopsy. To date there is no consensus regarding optimal treatment. Radiotherapy, chemotherapy and surgery are used in different association. We report the first case reported in literature managed with an urgent hysteroscopic resection of a primary cervical diffuse large B-cell lymphoma, followed by adjuvant radiotherapy. Relevant literature was reviewed. Our conservative approach needs to be validated in the future, especially for women with pregnancy desire and for those with low performance status. To date, after 24 months from diagnosis, our patient is still disease-free.

## 1. Introduction

The female genital tract can be involved as a secondary manifestation of disseminated lymphomas or leukemia, but is rarely the primary site of so-called extranodal lymphomas. Primary lymphomas of the female genital tract can affect the uterine corpus, uterine cervix, vulva, vagina or adnexa. 

From the early 1970s to the early 21st century, the incidence rates of non-Hodgkin lymphomas (NHL) nearly doubled in United States. Currently, NHLs account for around 4% of all new cancer diagnoses [1], and approximately one-third are extranodal. Around 0.5–1.5% of all extranodal NHLs arise from female genital tract and only 0.12–0.6% from the cervix. Nevertheless, only about 0.008% of all cervical tumors are primary malignant lymphomas [2,3,4,5,6,7,8,9].

In early stages, it is quite easy to define it as a primary cervical lymphoma since the disease is confined to the uterine cervix, while it is different in advanced disease due to diffuse lymphadenopathy or even bone marrow involvement. The strictest definition of primary extranodal NHL is “lymphoma presenting only in extra-nodal sites, with no visible lymphadenopathy on imaging” [10]; thus, only I E lymphomas are included (Ann Arbor staging). When the disease involves contiguous sites (e.g., cervix and uterine corpus or cervix and upper vagina), the site with the largest area of involvement is defined as the primary site [11]. Fox et al. proposed the criteria for primary ovarian lymphomas: tumor confined to regional lymph nodes or neighboring organs at diagnosis; no bone marrow involvement; lack of malignant cells in peripheral blood; any distant extra-ovarian disease must occur at least several months after the appearance of ovarian lesions [12]. These could be easily applied to any other site of the female genital tract [13,14].

A history of prolonged minor abnormal vaginal bleeding is the most common presenting symptom of primary cervical lymphoma. Heavy bleeding at presentation is rare, with only a few cases reported in literature [15,16,17]. “B” symptoms such as fever, fatigue, weight loss and night sweats are usually absent. Since postmenopausal vaginal bleeding or intermenstrual bleeding is a nonspecific symptom, the first diagnostic hypothesis falls on one of the more common female genital tract pathologies. Depending on the age range, this could be a gynecologic cancer such as cervical or endometrial one, or a benign condition such as fibroids, adenomyosis or endometriosis. A diagnostic pitfall to consider is that cervical cytology is negative most of the time. This reflects the peculiar behavior of lymphomas, which usually spread through cervical stroma leaving the epithelium intact at least in early stages, differently from cervical adenocarcinoma. Therefore, preoperative diagnosis requires deep cervical biopsy [7]. The most frequent histological subtype is diffuse large B-cell lymphoma (DLBCL) accounting for more than 70% of all cases [18,19].

To the best of the author’s knowledge, literature about primary lymphomas of the uterine cervix is limited to single case reports and limited case series. There are no greater studies or randomized controlled trials regarding diagnostic algorithms or treatment strategies. Hence, no published guidelines exist.

Considering the available literature, chemotherapy seems to be the cornerstone of cervical lymphoma management, alone or in combination with radiotherapy and/or surgical resection. This comes from the established treatment of nodal NHLs [20]. The most common surgical approach is total hysterectomy with or without salpingo-oophorectomy and lymphadenectomy. 

Prognosis of primary cervical lymphomas is usually excellent, with only a few cases of relapse or death reported [8,21,22] in the first five years after diagnosis, probably because diagnosis and treatment occur in early stages (I–II E).

The aim of our study is to present the first case of DLBCL (I E) of cervix uteri managed with an hysteroscopic approach followed by adjuvant radiotherapy. This is a starting point to validate in the future a new conservative and low-invasive treatment that is useful for those women who desire pregnancy or have a low performance status.

## 2. Case

An 83-year-old woman presented to our emergency department for a sudden copious vaginal bleeding without any other symptom. The woman was in good general conditions (ECOG Performance Status 0) with only hypertension and dyslipidemia in her past medical history. Considering her obstetric–gynecologic history, she had two vaginal spontaneous deliveries and a spontaneous menopause without any other reported atypical vaginal bleeding since she was 51 years old. The last Pap test, performed two years before, was reported negative. At time of presentation, her vital signs were all in normal range (blood pressure 130/70 mmHg, heart rate 90 bpm, Sat O_2_ 96%). 

Physical examination revealed no abnormal abdominal findings, no palpable cervical, axillary nor inguinal lymphadenopathy, nor hepatosplenomegaly. A thorough pelvic examination was difficult due to the retropubic position of the cervix, which was actively bleeding. The cervix appeared dilated by an exophytic bulky lesion partially protruding from external cervical orifice. Transvaginal echography revealed a bulky hypoechoic formation of the cervix of approximately 43 × 23.5 mm and color score 3. Corpus uteri seemed regular, with a thin endometrium (2.4 mm), and the ovaries were atrophic. During the exam the bleeding worsened, so an urgent hysteroscopy was performed to control the bleeding and obtain at least a biopsy of the mass. The procedure was carried on under general anesthesia without intubation. Hysteroscopy revealed the presence of an endocervical lesion of about 4 cm with a whitish appearance, fibrous consistency and accentuated vascularization.

Hysteroscopic resection was performed with monopolar electrodes using glycine 1.5% as irrigant solution. Removed tissue appeared friable and easily bleeding, with areas of cerebroid aspect. Cautious resection of the mass was performed until the internal uterine orifice was visualized. Continuing the hysteroscopy inside the uterus, an apparently regular uterine cavity with an atrophic endometrium was found. 

At the end of the procedure, hemostasis was achieved through electrocoagulation and iodoform gauze apposition.

The patient was dismissed after two days in good general conditions, with no pain, no vaginal bleeding and a reconstituted cervix. Blood tests were unremarkable.

**Figure 1:**
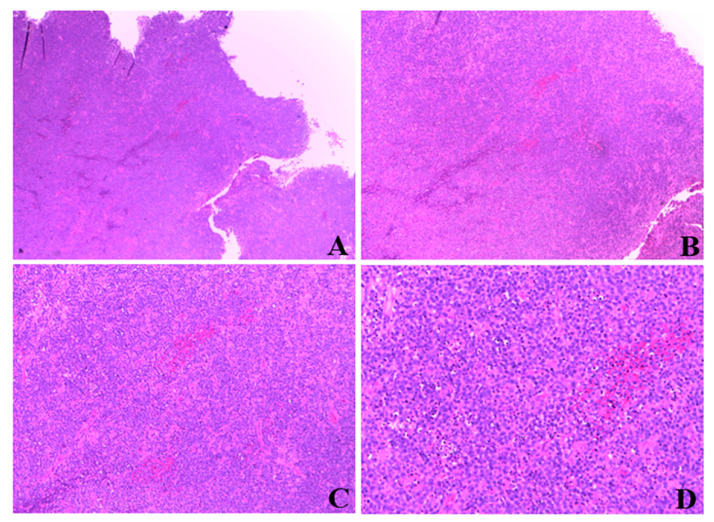
Magnification of cervical mass biopsy. (**A**): 2.5×. (**B**): 5×. (**C**): 10×. (**D**): 20×.

**Figure 2:**
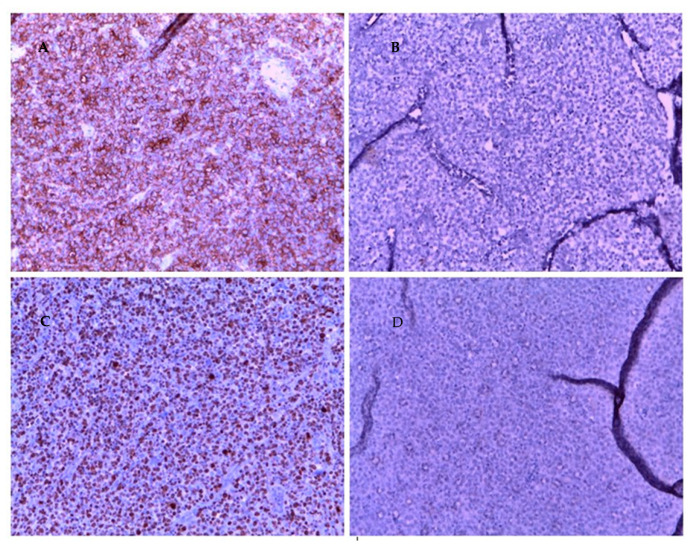
Immunohistochemistry of cervical mass biopsy, 20× magnification. (**A**): CD 20 positive. (**B**): CKAE1 AE3 negative. (**C**): high Ki 67. (**D**): SOX 10 neg.

Histologic examination of resected tissue showed a pleomorphic population of malignant lymphoid cells with a diffuse growing pattern and high mitotic index (Figure 1). Immunohistochemical study confirmed a CD20+, CD5+ germinal center-type diffuse large B cell lymphoma (Figure 2). Chest radiography, blood cell and differential count, renal and liver function, lactate dehydrogenase and echocardiography and FDG-PET/CT were all unremarkable. Given all the findings, a diagnosis of primary extranodal NHL of the cervix stage I E-A was made (modified Ann Arbor staging [23]). Considering patient age, neoplasia stage, the low risk of recurrence (calculated using the international prognostic index IPI) [24] and the almost complete surgical excision of the mass, only local radiotherapy (45 Gy/25 fr) was performed, reserving chemo/immunotherapy for an eventual future relapse.

The follow-up consists of hematological and gynecological examination every 3 month during the first 2 years, then every 6 months for the next 3 years, and annually thereafter to monitor for late relapse. In addition, a PET/TC control is performed every 6 months. To date, 20 months after diagnosis, the patient is still disease-free.

## 3. Discussion

As often happens for rare diseases, the therapeutic strategy is not uniform due to the lack of cases studied. 

What we know about lymphoma therapy is taken from the deep-rooted knowledge of nodal NHL treatment. In general, the cornerstone of treatment is chemotherapy; most reported cases are treated accordingly with chemotherapy alone or in association with radiotherapy [25]. Surgery is usually performed cautiously if a precise preoperative diagnosis is not possible. Usually, a radical surgery is performed with hysterectomy and bilateral salpingo-oophorectomy. Sometimes a more conservative approach is attempted with conization or trachelectomy [26]. In the published literature we had not found other cases of cervical lymphoma managed with hysteroscopic resection. There are just some reported cases of massive bleeding due to cervical lymphoma managed with emergent embolization of uterine arteries or emergent hysterectomy [15].

We tried a hysteroscopic approach mainly for two reasons: to rapidly achieve hemostasis and to obtain a biopsy of the mass. However, during the procedure, after a partial reduction in the lesion and hemostasis that allowed a better visualization, we identified a cleavage plan that made it technically possible to remove the whole mass [27,28].

At diagnosis, about 30% of women are under 40 years of age and likely desire pregnancy; another 15% are over 80 years old and should not be eligible for chemotherapy [29]. Taking this into account, an approach as conservative and noninvasive as possible is important to find out. 

To date, in young women who want to preserve their fertility the approach has been chemotherapy (CHOP—cyclophosphamide–doxorubicin–vincristine–prednisone chemotherapy regime) and immunotherapy to reduce the need for radiotherapy or surgical resection. The reported evidence regarding non-Hodgkin’s lymphoma in general suggests that in cases of childhood NHL, and in cases where fertility preservation is desired, current chemotherapeutic regimens are safe and can spare fertility, particularly when GnRH agonists are used in conjunction with treatment [30]. Other fertility-sparing techniques exist as ovarian transposition in those women who undergo RT [31]. To date, the conservative surgery of cervical DLBCL consists of conization or trachelectomy. However, pregnancy after trachelectomy is high-risk because of the increased rate of midtrimester miscarriage and preterm delivery, often as a consequence of preterm prelabor rupture of membranes [32]. Conceiving after conization is lower-risk, but the risk of preterm birth is increased compared to normal [33].

In patients who become pregnant after cervix uteri lymphoma, the mode of delivery should be well-discussed before childbirth, particularly if surgery or RT has been performed. RT does not seem to reduce the possibility of a safe vaginal delivery (in the literature, a radiation therapy with 30–6 Gy after ovarian transposition was reported) even if the safety of vaginal delivery after RT is still not adequately known [30]. 

For this reason, in young women considering future pregnancy, a hysteroscopic removal of the mass followed by RT could be preferred over conization or trachelectomy as a more conservative approach.

Even if avoidance of chemotherapy as a general rule cannot be stated, it will be important to study whether a local approach such as hysteroscopic surgery followed by adjuvant RT could be more effective and safer than a systemic approach with chemotherapy in the early stage of DLBCL of the cervix. A similar approach was described by Lee et al., who performed radiotherapy after radical surgery in two younger women [34]. In 1998, the same author reported the absence of recurrence in the two patients after a follow-up of ten years. 

In fact, avoiding systemic chemotherapy allows for the bypassing of its possible adverse effects such as anemia, diarrhea, nausea, hair loss, tiredness, mucositis, bladder irritation, neutropenia, infections, fluid retention and edema, appetite loss, stomach ache or indigestion, high blood sugar, numbness in hands or feet, jaw pain, dry skin, weakened nails, weight changes and vomiting [35]. Furthermore, choosing a conservative surgery protects from complications of major surgeries, such as bleeding and damage to nearby organs, as well as from those of general anesthesia. This lighter-touch approach could be more indicated also in those elderly women who would not be eligible for chemotherapy. 

Longer follow-up will be needed to better evaluate the long-term outcome of our patient. To date, after 24 months from diagnosis, she is still disease-free. 

## 4. Conclusions

Primary cervical lymphomas in early stages usually have excellent prognosis with any treatment combination (radiotherapy and/or chemotherapy and/or surgical resection); hence, the aim is to find out the most conservative and least invasive treatment approach [36]. In this report, we present the first case of DLBCL (I E) of cervix uteri managed with an hysteroscopic approach followed by adjuvant radiotherapy with success. There is evidently the need for a larger study—possibly a multicenter randomized controlled trial—to define a specific classification and the best management for this rare tumor in different age-range classes.

## Data Availability

The authors confirm that the data supporting the findings of this study are available within the article.
